# Meta‐analysis of microarray data to determine gene indicators involved in cisplatin resistance in non‐small cell lung cancer

**DOI:** 10.1002/cnr2.1970

**Published:** 2024-02-13

**Authors:** Somayeh Hashemi Sheikhshabani, Paratoo Modarres, Soudeh Ghafouri‐Fard, Zeinab Amini‐Farsani, Lavin Khodaee, Nasibeh Shaygan, Zahra Amini‐Farsani, Mir Davood Omrani

**Affiliations:** ^1^ Student Research Committee, Department of Medical Genetics Shahid Beheshti University of Medical Sciences Tehran Iran; ^2^ Department of Cell and Molecular Biology and Microbiology, Faculty of Science and Technology University of Isfahan Isfahan Iran; ^3^ Department of Medical Genetics Shahid Beheshti University of Medical Sciences Tehran Iran; ^4^ Department of Biotechnology and Plant Breeding Islamic Azad University Science and Research Branch Tehran Iran; ^5^ Bayesian Imaging and Spatial Statistics Group, Institute of Statistics Ludwig‐Maximilian‐Universität München Munich Germany; ^6^ Department of Statistics Lorestan University Khorramabad Iran; ^7^ Urogenital Stem Cell Research Center Shahid Beheshti University of Medical Sciences Tehran Iran

**Keywords:** BER, cisplatin resistance, EMT, microarray, non‐small cell lung cancer, PARP3

## Abstract

**Background:**

Lung cancer is a major cause of cancer‐related mortality worldwide, with a 5‐year survival rate of approximately 22%. Cisplatin is one of the standard first‐line chemotherapeutic agents for non‐small cell lung cancer (NSCLC), but its efficacy is often limited by the development of resistance. Despite extensive research on the molecular mechanisms of chemoresistance, the underlying causes remain elusive and complex.

**Aims:**

We analyzed three microarray datasets to find the gene signature and key pathways related to cisplatin resistance in NSCLC.

**Methods and Results:**

We compared the gene expression of sensitive and resistant NSCLC cell lines treated with cisplatin. We found 274 DEGs, including 111 upregulated and 163 downregulated genes, in the resistant group. Gene set enrichment analysis showed the potential roles of several DEGs, such as *TUBB2B*, *MAPK7*, *TUBAL3*, *MAP2K5*, *SMUG1*, *NTHL1*, *PARP3*, *NTRK1*, *G6PD*, *PDK1*, *HEY1*, *YTHDF2*, *CD274*, and *MAGEA1*, in cisplatin resistance. Functional analysis revealed the involvement of pathways, such as gap junction, base excision repair, central carbon metabolism, and Notch signaling in the resistant cell lines.

**Conclusion:**

We identified several molecular factors that contribute to cisplatin resistance in NSCLC cell lines, involving genes and pathways that regulate gap junction communication, DNA damage repair, ROS balance, EMT induction, and stemness maintenance. These genes and pathways could be targets for future studies to overcome cisplatin resistance in NSCLC.

## 1 INTRODUCTION

Lung cancer is one of the most common malignancies worldwide, with the greatest number of deaths in 2022, amounting to almost more than 350 deaths per day. Considering all stages, lung cancer has a 5‐year survival rate of approximately 22%.^1^ Non‐small cell lung cancer (NSCLC) is a heterogeneous subtype of the tumor, accounting for over 85% of all new lung cancer cases diagnosed.^2^

One of the standard clinical trials applied for the management of NSCLC is a regimen based on platinum (cisplatin or carboplatin), a cytotoxic chemotherapeutic agent.^3^, ^4^ However, the survival rate is poor in NSCLC due to the occurrence of drug resistance.^5^, ^6^

Cisplatin exerts cytotoxic effects by crosslinking with the purine bases on the DNA and interfering with DNA replication, DNA repair mechanisms, and cell division in cancer cells. Various signal transduction pathways are implicated in the cellular fate upon absorption of cisplatin into the cancer cell, including cisplatin‐induced oxidative stress, DNA damage, calcium signaling, cell apoptosis, activation/downregulation of mitogen‐activated protein kinase (MAPK), Jun amino‐terminal kinase (JNK), and phosphoinositide 3‐kinase (PI3K)‐Akt signaling pathways.^7^, ^8^

Despite the increasing prevalence of advanced therapeutics such as molecular targeted therapies to improve overall survival, drug resistance remains a frustrating, unsolved challenge in the management of cancer. Although a large number of studies have been dedicated to discovering the molecular events underlying the process of cancer drug resistance, the biological story behind drug resistance remains ambiguous, and the high mortality of lung cancer resulting from chemo‐resistance poses a significant ongoing clinical problem. In addition to the accumulation of genomic mutations, alterations at the transcriptomic levels during cancer progression can also be responsible for limiting the efficacy of anti‐tumor agents and the development of chemotherapy resistance. As a result, drug insensitivity of cancer cells may emerge at many levels, including the dysregulation of drug transporters, drug‐targeted agents, and pro‐survival and anti‐apoptotic molecules.^9^

Microarray technology is a high‐throughput approach used to detect the expression values of thousands of genes simultaneously and uncover differences in the transcriptome of cells and tissues.^10^ Accordingly, bioinformatics analysis of microarray data and system biology, including identification of gene signature, gene ontology, and pathway enrichment analysis, could be noteworthy to highlight potential key genes and pathways within a biological process.^11^ Therefore, microarray data analysis has been widely used in cancer pathogenesis and pharmacology research in recent years.

In the current report, we aimed to reveal the differentially expressed genes and underlying mechanisms associated with cisplatin resistance in NSCLC cell lines. This can help delineate biological subsets of resistant cancer cells and identify novel plausible therapeutic targets to overcome drug resistance. For this purpose, an integrative microarray analysis of non‐small cell lung cancer was conducted to compare the expression profiles of NSCLC cell lines under treatment with cisplatin, focusing on different responses to this anti‐tumor agent, whether sensitive (responder) or resistant (non‐responder). To reveal key genes and pathways involved in the development of cisplatin resistance, differentially expressed genes (DEGs) with statistical significance were subjected to gene and pathway enrichment analysis. Our results indicated that Epithelial‐mesenchymal transition (EMT) acts as a central regulator of cisplatin resistance, which is reinforced by several cross‐talking pathways.

## 2 METHOD

### 2.1 Integrative analysis of GEO datasets

Three microarray datasets were obtained from the National Center for Biotechnology Information‐Gene Expression Omnibus (NCBI‐GEO). These datasets contain the mRNA expression profiles of NSCLC samples that had received cisplatin antitumor agent treatment. The following key terms were searched in GEO to retrieve the datasets by filtering for “Expression profiling by array” and “Homo sapiens”: “Neoplasms/Carcinoma/Lung cancer/NSCLC” AND “Drug resistance/Recurrence/Relapse” AND “Cisplatin/CDDP/platinum‐based.” Samples were divided into two categories: Cisplatin‐sensitive and ‐resistant. All expression data were preprocessed, log2 transformed, and normalized using affy, gene filter, and limma Bioconductor packages provided in the R platform version 4.2.1 (https://www.r-project.org/).^12^, ^13^

For the comparative study and to remove unwanted variations across studies included, samples of datasets were integrated and batch corrections were conducted using an empirical Bayes (Combat) method from the sva package.^14^, ^15^ The logarithmic fold change between the two chemo‐sensitive and ‐resistant groups were calculated using a linear model procedure as implemented in the limma package.^12^ Principal component analysis (PCA), an unsupervised exploratory data analysis approach, was applied to control quality and visualize the variation between the arrays.^16^ After the elimination of sample arrays with poor quality and removal of batch effect, the *t*‐statistics test was applied to adjusted samples to evaluate differential expression gene values between cisplatin‐resistant and ‐sensitive NSCLC cells. The threshold of |logFC| ≥ 2 and *p.value* <.01 was considered to screen the significantly differentially expressed genes, and the Benjamini–Hochberg (BH) method was also used to reduce false positive values.^17^

### 2.2 Gene set enrichment analysis

The Enrichr online tool (https://maayanlab.cloud › Enrichr) was utilized to perform functional enrichment analysis of statistically significant DEGs. This analysis categorized and annotated the genes in terms of molecular function (MF), biological process (BP), and cellular component (CC).^18^ It also enriched the Kyoto Encyclopedia of Genes and Genomes (KEGG) pathways related to NSCLC resistance to cisplatin, an anti‐tumor agent. Gene Ontology (GO) and significant pathways enriched by differentially expressed genes (DEGs) using the Enrichr tool were considered significant with a *p.value* of <.05. The STRING database (https://www.string-db.org/) was utilized to assess protein–protein interaction (PPI) and identify hub genes involved in cisplatin resistance in NSCLC. The full STRING network option with a required high confidence of 0.700 was applied to construct the PPI network of differentially expressed genes. A functional enrichment analysis was also conducted to identify the over‐represented biological processes, pathways, and domains among the proteins in the network. The default FDR stringency of 0.05 was used for the enrichment analysis.

### 2.3 Receiver operating characteristic curve analysis

To assess the predictive values of identified DEGs in cisplatin resistance of NSCLC cells, the ROC curve was plotted for several key genes based on the gene expression data using GraphPad Prism 9.4.0 software. In the context of receiver operating characteristic (ROC) curve analysis, genes that demonstrate an Area Under the Curve (AUC) value exceeding 0.7, along with a statistically significant *p.value* of less than .01, are considered to exhibit a robust predictive potential for cisplatin resistance in NSCLC cells.

## 3 RESULTS

### 3.1 Microarray data analysis

Three datasets, GSE108214, GSE84146, and GSE21656, which contain expression profiles of cisplatin‐resistant and ‐sensitive lung cancer cell lines, were included in this study. The datasets were as follows: 1‐GSE108214 consists of expression profiling of 22 sensitive and resistant non‐small cell lung cancer cells (A549) that were treated with 11 or 34 μM cisplatin or with the drug‐free medium, 2‐GSE84146 involved gene expression profiles of two paired cisplatin‐sensitive and ‐resistant lung cancer cell lines (H460, H23), 3‐GSE21656 encompassed expression profiling of cisplatin‐resistant lung cancer cells derived from the H460 lung cell line and the parental cells. The detailed information of selected datasets is presented in Table 1.

**TABLE 1:** Details of the microarray data used.

Dataset	Platform	Gene number	Tissue	Total sample	Involved samples	
					Cell types	Sample number
GSE108214	Agilent (GPL17077)	16 174	Lung	22	A549 cell line, Cisplatin **Sensitive**, control	4
					A549 cell line, Cisplatin **Sensitive**, 24 h cisplatin, 11 μM	3
					A549 cell line, Cisplatin **Resistance**, control	5
					A549 cell line, Cisplatin **Resistance**, 24 h cisplatin, 11 μM	5
					A549 cell line, Cisplatin **Resistance**, 24 h cisplatin, 34 μM	5
GSE84146	Agilent (GPL6480)	12 443	Lung	12	Cisplatin **Resistant** H23 lung cancer cell line	2
					Cisplatin **Resistant** H460 lung cancer cell line	2
GSE21656	Affymetrix (GPL6244)	19 382	Lung	6	Cisplatin **Resistant** cell derived from H460	3

**FIGURE 1:**
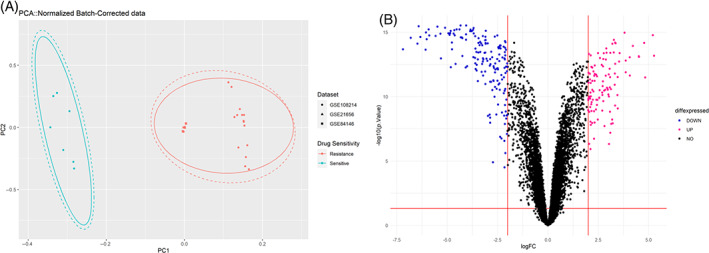
The principal component analysis (PCA) and volcano plot. (A) The PCA plot was conducted based on the normalized integrated expression data. Clustering the samples into two distinct groups using this unsupervised approach demonstrates the removal of unwanted variations among datasets. (B) The volcano plot for differentially expressed genes (DEGs) in cisplatin‐resistant NSCLC cell lines versus sensitive ones shows the fold‐change (*x*‐axis) versus the significance (−log10 (*p.value*) on the *y*‐axis) of the identified DEGs. The significant DEGs were identified based on *p.value* <.01 and |logFC| > 2. Two vertical lines show the 2‐fold change boundaries and the horizontal line shows the cutoff of statistical significance (*p.value* ≤ .01). Pink and blue dots display upregulated and downregulated genes, respectively.

**FIGURE 2:**
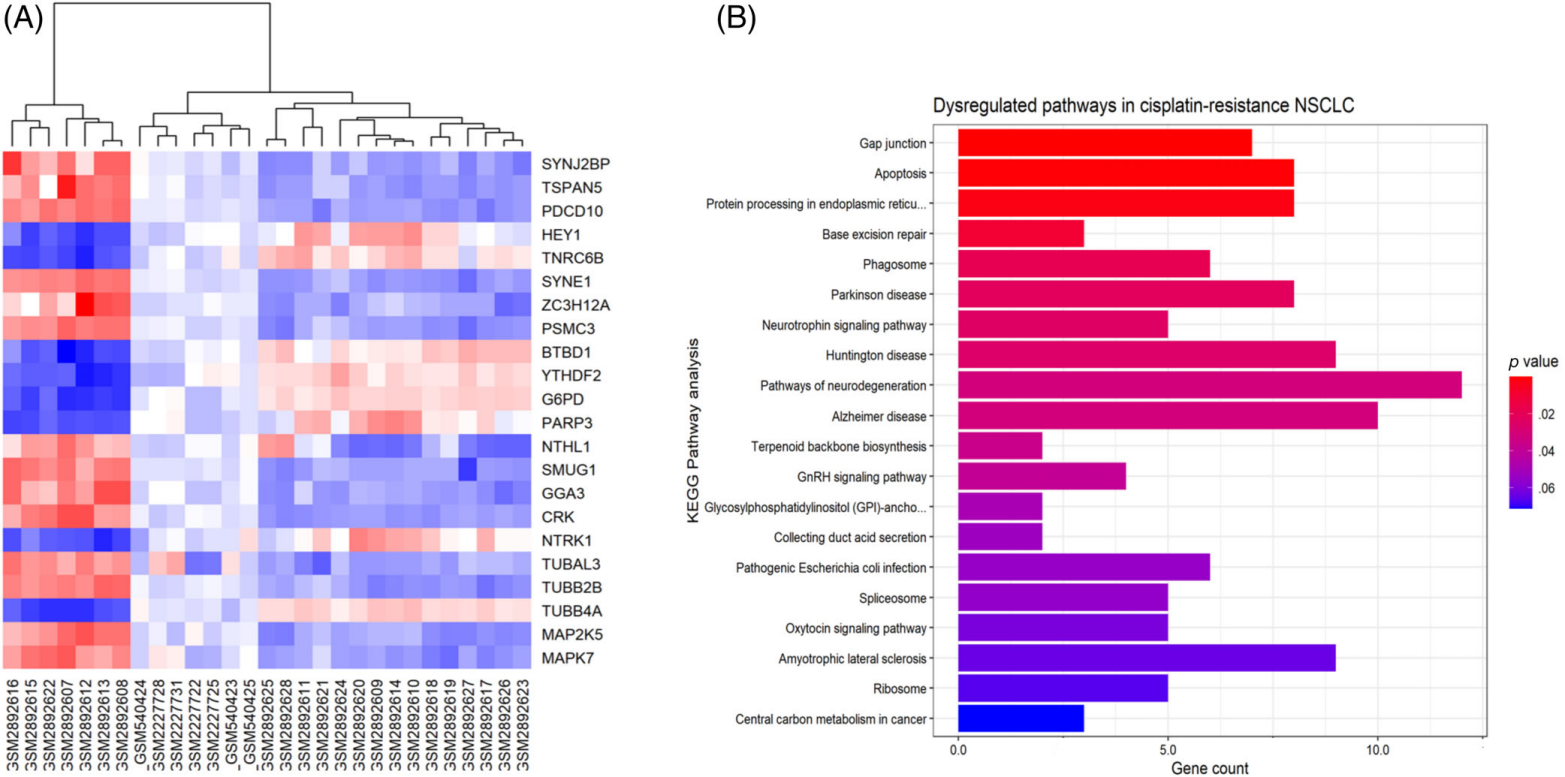
Heatmap and KEGG pathway analysis of differentially expressed genes. (A) The heatmap provides a visual representation of the varying levels of gene expression in cisplatin‐sensitive and ‐resistant NSCLC cell lines. High expression is denoted by the color red, while low expression is indicated by blue (S represents sensitive; R represents resistance). (B) The bar plot presents the 20 most representative altered canonical KEGG pathways that are affected by the differentially expressed genes (DEGs) associated with cisplatin resistance in NSCLC. Each term's statistical significance was evaluated using a *p.value* <.05, with the most significantly enriched pathways depicted in a progressively redder color.

A total of 29 samples, consisting of 7 responder cells to cisplatin, referred to as sensitive cells in this study, and 22 cisplatin‐resistant lung cancer cell lines, were selected for further analysis. The expression values of GSE108214, GSE84146, and GSE21656 were integrated based on common Entrez gene IDs. Figure 1A shows the PCA plot visualizing samples as two clusters, cisplatin‐resistant or ‐sensitive, based on overall patterns of the expression signatures and supports the removal of batch effect. Among 10 667 common genes in all three datasets, 274 DEGs were significantly identified by considering *p.value* <.01 and |logFC| ≥ 2 as a threshold, consisting of 111 upregulated genes and 163 downregulated genes associated with cisplatin resistance (Tables 2, 3, respectively). Figure 1B shows the volcano plot for differentially expressed genes between cisplatin‐resistant and ‐sensitive NSCLC cell lines. The expression value of each gene in both cisplatin‐resistant and ‐sensitive samples are provided in Table S2. These levels are also visually represented through a heatmap, which was created using unsupervised hierarchical cluster analysis (Figure 2A).

**TABLE 2:** Upregulated genes in Cisplatin‐resistant NSCLC.

Gene ID	Gene symbol	logFC	*p*.value	adj.*p*.val	Gene ID	Gene symbol	logFC	*p*.value	adj.*p*.val
79663	HSPBAP1	5.274432	6.77E–14	7.37E–12	84922	FIZ1	2.710456	7.48E–11	2.21E–09
9968	MED12	5.210812	1.74E–15	6.57E–13	23462	HEY1	2.700349	1.23E–09	2.50E–08
30827	CXXC1	4.830429	3.29E–12	1.51E–10	91782	CHMP7	2.69949	4.93E–11	1.54E–09
84981	MIR22HG	4.782434	6.86E–15	1.38E–12	64374	SIL1	2.674849	3.44E–13	2.46E–11
81930	KIF18A	4.52592	8.31E–14	8.60E–12	11221	DUSP10	2.586421	1.03E–13	9.98E–12
23510	KCTD2	4.515531	7.45E–14	7.87E–12	79132	DHX58	2.569289	3.16E–11	1.07E–09
2081	ERN1	4.219453	1.18E–14	1.93E–12	1509	CTSD	2.552749	1.28E–10	3.51E–09
26471	NUPR1	4.068072	2.34E–12	1.14E–10	122416	ANKRD9	2.551555	1.20E–10	3.32E–09
84279	PRADC1	4.064377	8.83E–14	8.97E–12	340719	NANOS1	2.488622	4.24E–14	4.92E–12
57461	ISY1	3.866154	1.30E–12	6.92E–11	10318	TNIP1	2.478066	6.10E–09	9.99E–08
26297	SERGEF	3.865079	1.60E–13	1.38E–11	55216	NKAPD1	2.464131	1.80E–10	4.71E–09
11059	WWP1	3.810716	1.09E–15	6.04E–13	3659	IRF1	2.438822	4.47E–10	1.04E–08
55092	TMEM51	3.559023	4.21E–14	4.92E–12	83541	FAM110A	2.437903	2.69E–12	1.28E–10
56904	SH3GLB2	3.536627	1.92E–13	1.58E–11	58985	IL22RA1	2.408778	1.77E–10	4.67E–09
57683	ZDBF2	3.473829	8.42E–10	1.81E–08	123016	TTC8	2.398783	1.99E–12	9.96E–11
55272	IMP3	3.467123	3.03E–12	1.42E–10	116068	LYSMD3	2.388382	9.99E–10	2.10E–08
10594	PRPF8	3.451994	7.66E–11	2.26E–09	22859	ADGRL1	2.330797	1.75E–12	8.88E–11
10382	TUBB4A	3.361404	7.33E–15	1.39E–12	22818	COPZ1	2.327023	3.83E–13	2.71E–11
10212	DDX39A	3.34342	2.84E–13	2.12E–11	25896	INTS7	2.323668	1.04E–08	1.62E–07
5912	RAP2B	3.339518	5.04E–10	1.14E–08	199745	THAP8	2.323617	6.06E–12	2.67E–10
3364	HUS1	3.335418	6.49E–15	1.38E–12	113457	TUBA3D	2.30119	2.44E–12	1.18E–10
7525	YES1	3.323891	1.62E–12	8.40E–11	2907	GRINA	2.294773	1.86E–07	1.96E–06
116362	RBP7	3.322187	1.22E–11	4.80E–10	54867	TMEM214	2.292595	1.38E–09	2.76E–08
29984	RHOD	3.277315	3.96E–15	9.78E–13	55301	OLAH	2.260619	3.99E–11	1.29E–09
4627	MYH9	3.272625	3.74E–14	4.75E–12	25797	QPCT	2.260285	1.11E–12	6.08E–11
29927	SEC61A1	3.223199	1.20E–08	1.82E–07	78988	MRPL57	2.234584	1.78E–10	4.67E–09
55297	CCDC91	3.207522	7.92E–15	1.42E–12	8303	SNN	2.209747	5.68E–13	3.56E–11
8796	SCEL	3.19112	6.06E–09	9.99E–08	339745	SPOPL	2.208911	3.00E–11	1.03E–09
64693	CTAGE1	3.145123	1.31E–08	1.94E–07	53339	BTBD1	2.20489	5.98E–12	2.65E–10
2034	EPAS1	3.132338	2.69E–11	9.38E–10	55572	FOXRED1	2.201267	1.12E–08	1.71E–07
283254	HARBI1	3.104806	4.04E–14	4.79E–12	160428	ALDH1L2	2.173372	2.71E–10	6.69E–09
7263	TST	3.101123	6.54E–08	7.88E–07	23640	HSPBP1	2.166988	1.31E–09	2.63E–08
138429	PIP5KL1	3.09283	3.13E–12	1.46E–10	1647	GADD45A	2.15773	4.97E–13	3.25E–11
2272	FHIT	3.083641	1.52E–09	2.99E–08	4103	MAGEA4	2.153113	3.95E–10	9.37E–09
4900	NRGN	3.077222	1.94E–11	7.29E–10	10179	RBM7	2.137409	1.58E–08	2.28E–07
51009	DERL2	3.068526	6.95E–13	4.14E–11	10435	CDC42EP2	2.133609	9.60E–11	2.77E–09
6192	RPS4Y1	3.038442	4.83E–07	4.50E–06	22894	DIS3	2.130237	8.90E–12	3.72E–10
84557	MAP1LC3A	3.029246	9.76E–11	2.79E–09	150094	SIK1	2.129382	6.28E–14	6.93E–12
84245	MRI1	3.024615	1.89E–12	9.49E–11	6262	RYR2	2.116523	4.95E–11	1.54E–09
116442	RAB39B	3.005068	6.85E–11	2.05E–09	202459	OSTCP1	2.114287	9.01E–09	1.42E–07
6691	SPINK2	2.974462	2.88E–11	9.98E–10	5163	PDK1	2.114258	1.19E–06	9.95E–06
5718	PSMD12	2.959607	2.18E–11	7.97E–10	10039	PARP3	2.105644	6.53E–10	1.43E–08
26093	CCDC9	2.933826	2.08E–11	7.65E–10	2815	GP9	2.100169	1.44E–10	3.88E–09
90060	CCDC120	2.916987	4.00E–14	4.79E–12	51367	POP5	2.090686	4.75E–12	2.15E–10
2539	G6PD	2.901803	1.82E–14	2.72E–12	23112	TNRC6B	2.08547	1.72E–12	8.76E–11
57215	THAP11	2.875863	1.34E–13	1.16E–11	23678	SGK3	2.073435	1.33E–10	3.62E–09
57610	RANBP10	2.874541	2.39E–12	1.16E–10	83479	DDX59	2.055844	1.01E–10	2.88E–09
51552	RAB14	2.855128	2.95E–11	1.02E–09	261726	TIPRL	2.055022	1.55E–09	3.05E–08
5926	ARID4A	2.809923	1.95E–08	2.72E–07	4801	NFYB	2.050887	4.74E–07	4.43E–06
128	ADH5	2.793516	3.41E–11	1.13E–09	28992	MACROD1	2.038955	4.95E–14	5.62E–12
80349	SKIC8	2.774198	4.34E–14	4.98E–12	51441	YTHDF2	2.022422	1.43E–12	7.49E–11
8518	ELP1	2.754307	1.22E–13	1.11E–11	7580	ZNF32	2.017997	8.27E–12	3.52E–10
4086	SMAD1	2.750286	2.25E–10	5.70E–09	535	ATP6V0A1	2.015482	1.43E–10	3.88E–09
83940	TATDN1	2.737472	4.55E–13	3.07E–11	7188	TRAF5	2.010007	1.84E–10	4.78E–09
1893	ECM1	2.733292	6.51E–12	2.82E–10	4914	NTRK1	2.00152	1.15E–09	2.35E–08
80152	CENPT	2.719131	1.57E–11	6.06E–10					

**TABLE 3:** Downregulated genes in Cisplatin‐resistant NSCLC.

Gene ID	Gene symbol	logFC	*p*.value	adj.*p*.val	Gene ID	Gene symbol	logFC	*p*.value	adj.*p*.val
64793	CEP85	–2.0157	2.67E–09	4.78E–08	81628	TSC22D4	–2.40423	1.34E–14	2.13E–12
220972	MARCHF8	–2.01644	4.96E–13	3.25E–11	5569	PKIA	–2.4054	9.51E–12	3.91E–10
10073	SNUPN	–2.01848	2.69E–13	2.05E–11	23481	PES1	–2.41093	1.75E–13	1.46E–11
113452	TMEM54	–2.03197	8.03E–08	9.33E–07	171483	FAM9B	–2.41206	5.43E–14	6.10E–12
10331	B3GNT3	–2.03283	1.09E–12	6.04E–11	84893	FBH1	–2.44383	1.79E–14	2.72E–12
79861	TUBAL3	–2.04648	2.98E–09	5.28E–08	64940	STAG3L4	–2.44847	1.15E–11	4.56E–10
60436	TGIF2	–2.06238	1.71E–10	4.56E–09	1798	DPAGT1	–2.45837	1.46E–14	2.30E–12
11034	DSTN	–2.06691	5.04E–09	8.48E–08	9263	STK17A	–2.47377	4.19E–10	9.82E–09
79921	TCEAL4	–2.07202	4.76E–13	3.19E–11	1365	CLDN3	–2.48124	7.80E–13	4.57E–11
3422	IDI1	–2.07713	2.34E–14	3.24E–12	23335	WDR7	–2.4901	6.29E–13	3.86E–11
6939	TCF15	–2.09172	1.43E–09	2.83E–08	55333	SYNJ2BP	–2.50468	5.93E–12	2.63E–10
5598	MAPK7	–2.10173	1.48E–12	7.69E–11	127544	RNF19B	–2.51093	1.79E–15	6.57E–13
29781	NCAPH2	–2.10599	6.15E–15	1.37E–12	3420	IDH3B	–2.51575	1.22E–13	1.11E–11
50862	RNF141	–2.10887	2.47E–06	1.87E–05	3213	HOXB3	–2.52886	6.67E–13	4.02E–11
23583	SMUG1	–2.11628	6.30E–14	6.93E–12	23157	SEPTIN6	–2.54494	2.50E–11	8.88E–10
10403	NDC80	–2.11803	2.55E–11	9.02E–10	58491	ZNF71	–2.55412	2.48E–09	4.48E–08
3104	ZBTB48	–2.12061	1.65E–14	2.55E–12	6209	RPS15	–2.60801	7.93E–13	4.62E–11
146059	CDAN1	–2.12603	4.56E–10	1.05E–08	140606	SELENOM	–2.61344	2.67E–13	2.05E–11
93621	MRFAP1	–2.12956	4.96E–15	1.15E–12	5089	PBX2	–2.62285	1.32E–12	7.01E–11
284013	VMO1	–2.13341	1.35E–08	1.99E–07	84936	ZFYVE19	–2.62639	1.78E–11	6.77E–10
10723	SLC12A7	–2.13425	3.55E–14	4.61E–12	2909	ARHGAP35	–2.6338	2.20E–15	7.11E–13
83591	THAP2	–2.1358	1.31E–13	1.15E–11	23264	ZC3H7B	–2.6486	1.03E–10	2.90E–09
55030	FBXO34	–2.14549	3.54E–11	1.17E–09	4673	NAP1L1	–2.67738	5.91E–13	3.68E–11
6596	HLTF	–2.14894	5.94E–13	3.68E–11	388394	RPRML	–2.6962	6.14E–12	2.70E–10
6853	SYN1	–2.15242	3.09E–05	0.000172	84817	TXNDC17	–2.69687	4.41E–13	3.03E–11
55750	AGK	–2.15419	1.10E–13	1.03E–11	1891	ECH1	–2.70654	5.50E–12	2.46E–10
6845	VAMP7	–2.15671	2.75E–07	2.76E–06	4913	NTHL1	–2.72506	1.25E–05	7.73E–05
80149	ZC3H12A	–2.16844	3.04E–09	5.37E–08	10098	TSPAN5	–2.75422	3.11E–11	1.05E–09
56259	CTNNBL1	–2.184	3.19E–12	1.47E–10	7049	TGFBR3	–2.76494	8.93E–14	8.99E–12
84302	PGAP4	–2.19069	5.48E–11	1.69E–09	64093	SMOC1	–2.81044	3.33E–14	4.54E–12
5873	RAB27A	–2.19149	1.33E–14	2.13E–12	6749	SSRP1	–2.81506	7.17E–12	3.07E–10
7337	UBE3A	–2.25765	9.35E–14	9.24E–12	114659	LRRC37B	–2.84778	3.10E–13	2.26E–11
10654	PMVK	–2.27239	4.69E–12	2.13E–10	23091	ZC3H13	–2.86348	1.06E–08	1.63E–07
28977	MRPL42	–2.27766	1.71E–09	3.32E–08	84823	LMNB2	–2.86524	1.26E–13	1.13E–11
50615	IL21R	–2.30371	1.80E–07	1.90E–06	6319	SCD	–2.86633	4.35E–09	7.44E–08
10484	SEC23A	–2.30974	2.12E–12	1.05E–10	6102	RP2	–2.8683	7.06E–12	3.04E–10
8225	GTPBP6	–2.34409	1.10E–14	1.83E–12	7597	ZBTB25	–2.88403	4.07E–13	2.84E–11
80267	EDEM3	–2.34603	1.84E–14	2.72E–12	64793	CEP85	–2.0157	2.67E–09	4.78E–08
9718	ECE2	–2.36967	1.34E–07	1.48E–06	220972	MARCHF8	–2.01644	4.96E–13	3.25E–11
9746	CLSTN3	–2.37489	1.08E–14	1.83E–12	10073	SNUPN	–2.01848	2.69E–13	2.05E–11
25824	PRDX5	–2.38932	2.96E–11	1.02E–09					

### 3.2 Gene set enrichment analysis

The enrichment analysis of KEGG pathways suggests a potential correlation between the differentially expressed genes (DEGs) and drug resistance via multiple pathways. Figure 2B illustrates the top 20 enriched pathways. Pathways that were most impacted, exhibiting a statistical significance of *p.value* <.05, are represented in the red spectrum. Among them, the most significant pathways were the gap junction, apoptosis, protein processing in the endoplasmic reticulum, base excision repair, phagosome, neurotrophin signaling pathway, pathways of neurodegeneration, terpenoid backbone biosynthesis, GnRH signaling pathway, and glycosylphosphatidylinositol (GPI)‐anchor biosynthesis. Our analysis revealed that seven dysregulated genes (*TUBB2B*, *TUBA3D*, *MAPK7*, *TUBAL3*, *ADCY1*, *TUBB4A*, and *MAP2K5*) are potentially implicated in the gap junction pathway, which is posited to be one of the most critical pathways in this context.

**FIGURE 3:**
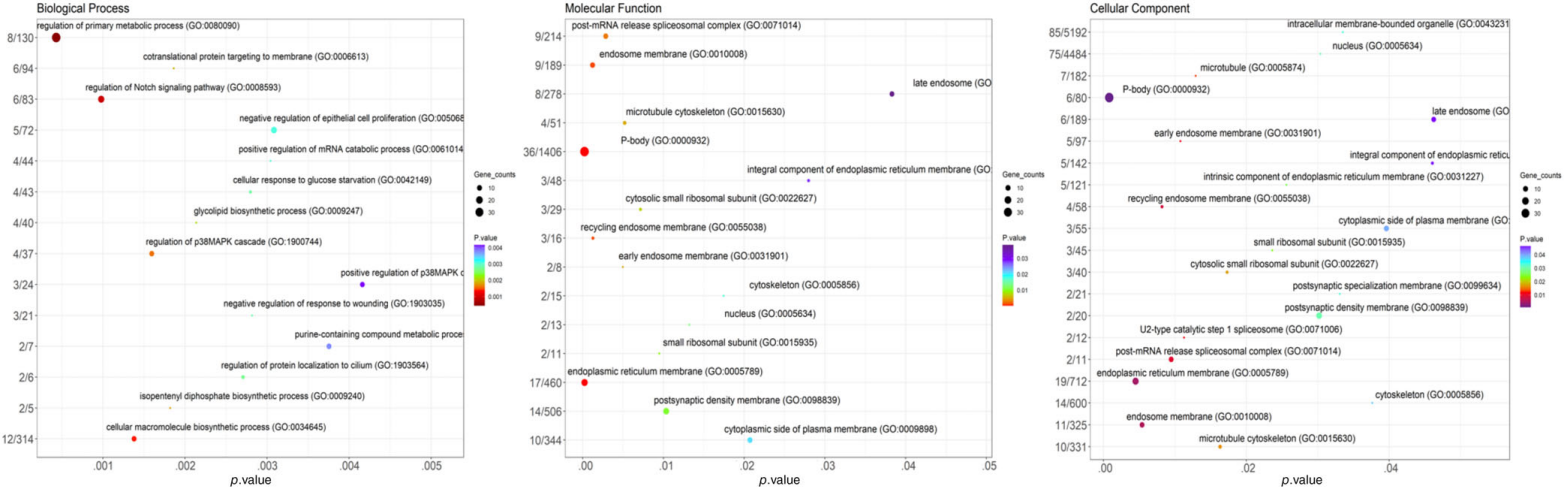
Gene Ontology (GO) of DEGs. The gene ontology of differentially expressed genes (DEGs) reveals the top 20 terms in three categories: biological process (BP), molecular function (MF), and cellular component (CC). The color of the bubbles ranges from red to blue, indicating a progression from smaller to larger *p.value*. The size of the bubbles corresponds to the number of genes, with larger bubbles representing a greater number of genes.

The Gene Ontology term enrichment analysis has provided a comprehensive understanding of the probable functions of differentially expressed genes (DEGs) in the development of chemotherapy resistance in NSCLC cells. Through the application of Gene Ontology (GO) term enrichment analysis, genes differentially expressed in relation to cisplatin resistance were categorized into three functional groups: biological process (BP), cellular component (CC), and molecular function (MF). These categories are depicted in Figure 3 and further detailed in Tables [Link], [Link].

The biological process group includes several significant processes such as the biosynthesis of phospholipids (GO:0008654), cellular macromolecules (GO:0034645), and the cellular response to glucose starvation (GO:0042149). It also encompasses the regulation of multiple signaling pathways involved in primary metabolic processes (GO:0080090), including the Notch (GO:0008593) and p38MAPK (GO:1900744) cascades, as well as protein deubiquitination (GO:0090085). The cellular component group primarily consists of the P‐body, endoplasmic reticulum membrane, recycling endosome membrane, microtubule cytoskeleton, and cytosolic small ribosomal subunit. In the molecular function group, the most significant enrichments were found in purine ribonucleotide binding and protein binding.

**FIGURE 4:**
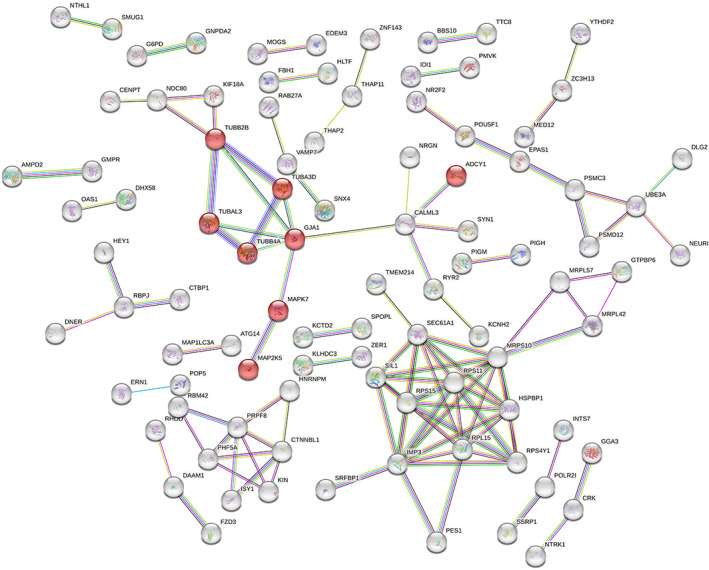
Protein–protein interaction network of DEGs. This figure illustrates the network of differentially expressed genes (DEGs) with potential roles in cisplatin resistance in NSCLC cells, constructed using the STRING database (version 12.0). The nodes represent proteins, and the edges denote interactions. The red nodes represent proteins encoded by seven DEGs (*TUBB2B*, *MAPK7*, *TUBA3D*, *TUBB4A*, *TUBAL3*, *ADCY1*, and *MAP2K5*) selected for their roles in the gap junction pathway. This pathway was the only one significantly over‐represented among the proteins in the network. The module reveals the connection between these DEGs and connexin43 (GJA1 gene), a key effector in the gap junction pathway. Notably, *GJA1* was not identified among the DEGs contributing to cisplatin‐resistant NSCLC.

Figure 4 illustrates the protein–protein interaction network and module selection, which were constructed utilizing the STRING database with a false discovery rate (FDR) stringency of 0.05. The functional enrichment analysis pinpointed a singular pathway, namely the gap junction (hsa04540), as being significantly over‐represented among the proteins within the network, with an FDR of 0.0165.

In order to evaluate the potential involvement of seven differentially expressed genes (DEGs)—*TUBB2B*, *TUBA3D*, *MAPK7*, *TUBAL3*, *ADCY1*, *TUBB4A*, and *MAP2K5*—within the gap junction pathway, a module was assembled. This module incorporated an additional gene, *connexin43* (*GJA1*), known for its pivotal role within the gap junction pathway. The module associated with the gap junction pathway has been denoted by the red nodes.

### 3.3 ROC curve analysis

**FIGURE 5:**
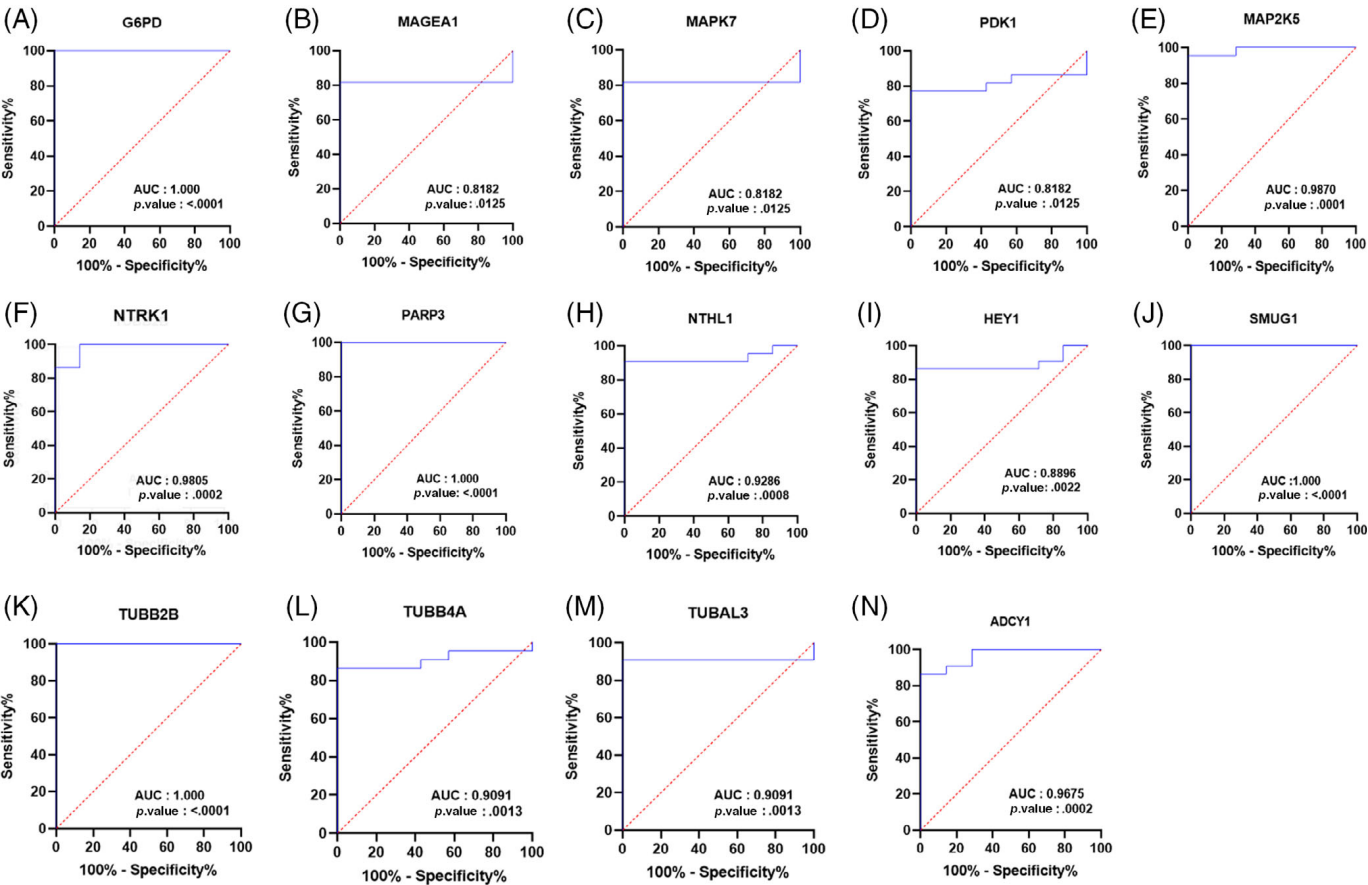
Receiver operating characteristic (ROC) analysis for DEGs. ROC plots illustrate the predictive accuracy of 14 DEGs in relation to the occurrence of cisplatin resistance in NSCLC cell lines. AUC refers to the area under the ROC curve. (A–N) are ROC plots of G6PD, MAGEA1, MAPK7, PDK1, MAP2K5, NTRK1, PARP3, NTHL1, HEY1, SMUG1, TUBB2B, TUBB4A, TUBAL3 and ADCY1 genes, respectively.

ROC curves were generated for 14 key genes, including *MAPK7*, *NTRK1*, *TUBB2B*, *MAP2K5*, *TUBB4A*, *SMUG1*, *PARP3*, *NTHL1*, *G6PD*, *TUBAL3*, *ADCY1*, *PDK1*, *HEY1*, and *MAGEA1*. As shown in Figure 5, all of these 14 DEGs demonstrated a desirable AUC ≥0.8 with a significant *p*‐value ≤.01.

## 4 DISCUSSION

Cisplatin, a widely used first‐line chemotherapeutic agent for advanced NSCLC, has been the focus of extensive clinical trials over the past few decades. Despite these efforts, the high mortality rate of lung cancer due to chemoresistance remains a significant clinical challenge.

In this study, we aimed to investigate the prognostic implications of cisplatin responsiveness in NSCLC cells. We conducted an integrative microarray analysis to identify dysregulated genes and key pathways involved in the development of resistance to the cisplatin antitumor agent. This analysis revealed a total of 274 genes with significant differences in expression, including 111 upregulated genes and 163 downregulated genes in cisplatin‐resistant NSCLC cell lines compared with a responder/sensitive group.

The functional analysis enriched several dysregulated signaling pathways associated with the occurrence of cisplatin resistance in NSCLC cell lines. These pathways include the Gap Junction, Protein Processing in the Endoplasmic Reticulum, Base Excision Repair, Phagosome, Terpenoid Backbone Biosynthesis, Glycosylphosphatidylinositol (GPI)‐anchor Biosynthesis, Neurodegeneration, GnRH, Neurotrophin, and Notch signaling pathways.

Among the DEGs, five downregulated genes (*TUBB2B* (logFC: −4.76441, *p.value*: 6.36E‐15), *MAPK7* (logFC: −2.10173, *p.value*: 1.48E‐12), *TUBAL3* (logFC: −2.04648, *p.value*: 2.98E‐09), *ADCY1* (logFC: −6.0183, *p.value*: 3.52E‐15) and *MAP2K5* (logFC: −5.30811, *p.value*: 1.10E‐13)), and two upregulated genes, *TUBA3D* (logFC: 2.30119, *p.value*: 2.44E‐12) and *TUBB4A* (logFC: 3.361404, *p.value*: 7.33E‐15) were found to be involved in gap junction pathways associated with cisplatin‐resistant NSCLC cell lines.

Several studies have shown that the deficiency of gap junction activity, which is influenced by the levels of connexins and tubulins, contributes to the resistance to chemotherapeutic agents, such as cisplatin, in various cancers, such as breast, colorectal, and lung.^19^, ^20^, ^21^, ^22^, ^23^, ^24^

Connexins are a group of transmembrane proteins that form gap junctions, which are intercellular channels that facilitate the exchange of small molecules and potentially control cellular growth and differentiation processes.^25^, ^26^

It has been reported that the expression and phosphorylation of Connexin43 (Cx43) can affect the epithelial‐mesenchymal transition (EMT) and the cisplatin sensitivity of A549 cell lines.^23^ This is because Cx43 is modified by mitogen‐activated protein kinases (MAPK), a group of proteins that can control gap junction intercellular communication (GJIC) by phosphorylating Cx43.^20^, ^27^, ^28^

As shown in Figure 4, a network of interactions between proteins TUBB2B, MAPK7, TUBAL3, MAP2K5, and GJA1 (Cx43) suggests that reduced expression of MAPK and tubulin genes could be responsible for the attenuation of the gap junction pathway and insensitivity of lung cancer cells to cisplatin.

Consistent with our hypothesis, a recent study has interestingly highlighted the importance of gap junction activation and MAPK pathways for overcoming cisplatin resistance in NSCLC.^29^ Huang et al. have reported a novel adjuvant compound, arteannuin B (Art B), that enhances the cytotoxicity of cisplatin by facilitating its uptake into the cancer cells. This is mediated by the upregulation of connexin43, the activation of gap junction and MAPK pathways, and the increase of intracellular Fe2+ and calcium influx.^29^

DNA damage, a common feature of many anticancer drugs, leads to genome instability and cell death in cancer cells treated with cisplatin. The base excision repair (BER) pathway modulates the sensitivity of cancer cells to cisplatin by repairing some of the DNA lesions induced by the drug. However, BER can also impair the efficiency of other DNA repair pathways, such as the nucleotide excision repair (NER) and homologous recombination (HR), that remove the interstrand cross‐links (ICLs) generated by cisplatin.

Functional analysis showed that *SMUG1* (logFC: −2.11628, *p.value*: 6.30E‐14), *PARP3* (logFC: 2.105644, *p.value*: 6.53E‐10), and *NTHL1* (logFC: −2.72506, *p.value*: 1.25E‐05) were the dysregulated genes in the base excision repair (BER) pathway, which could modulate chemoresistance in NSCLC. *NTHL1* and Single‐strand‐selective monofunctional uracil‐DNA glycosylase 1 (*SMUG1*) encoded enzymes that had crucial roles in the DNA base excision repair (BER) pathway. Consistent with these data, the downregulation of *NTHL1* and *SMUG1* might solve the problem of chemoresistance to cisplatin in lung cancer cells, but this finding required further experimental validation.

Our study reveals that *PARP3* expression is elevated in NSCLC cells that exhibit cisplatin resistance. According to previous studies, PARP3 participates in the ROS signaling pathway triggered by TGFβ, which promotes EMT and stemness in cancer cells.^30^ PARP3 also regulates the expression of TG2, an enzyme that influences EMT and stemness, by altering the chromatin state of TGFβ‐responsive genes.^30^ Interestingly, TGFβ has been shown to confer EMT‐mediated chemoresistance in NSCLC cell lines treated with cisplatin.^31^ In addition, PARP3 enhances the DNA repair proficiency and genomic stability of cancer cells by supporting the NHEJ process.^30^ Therefore, our study corroborates the importance of PARP3 upregulation in the development of cisplatin resistance in NSCLC. We suggest that PARP3 could be a potential therapeutic target for reversing EMT‐mediated chemoresistance in NSCLC.

Our finding also shows that *PARP3* is highly expressed in NSCLC cells that are resistant to cisplatin. PARP3 is involved in the ROS signaling pathway activated by TGFβ, which leads to EMT and stemness in cancer cells.^30^ PARP3 also controls the TG2 expression, an enzyme that plays a role in EMT and stemness, by changing the chromatin state of TGFβ‐responsive genes. Interestingly, TGFβ is involved in EMT‐mediated chemoresistance in NSCLC cell lines exposed to cisplatin.^31^ Furthermore, PARP3 improves the DNA repair ability and genomic stability of cancer cells by assisting the NHEJ process.^30^ Therefore, our finding supports the relevance of upregulated *PARP3* and the acquisition of cisplatin drug resistance. PARP3 could be potential target for anticancer therapies and its inhibiting may increase the effectiveness of cancer treatment by reversing the EMT‐mediated chemoresistance in NSCLC.

Metabolic reprogramming is a key feature of tumors that enables them to resist first‐line chemotherapy drugs.^32^, ^33^ One of the main pathways involved in this process is the central carbon metabolism, which includes the pentose phosphate pathway (PPP) and the tricarboxylic acid (TCA) cycle.^34^ These pathways support cancer growth and survival by providing energy and biosynthetic precursors.^32^, ^35^, ^36^ In the present study, we identified two genes related to central carbon metabolism, *G6PD* and *PDK1*, that were overexpressed in cisplatin‐resistant NSCLC cell lines. G6PD and PDK1 are known to regulate the Warburg Effect, a phenomenon in which cancer cells preferentially use glycolysis for energy production.^36^, ^37^ G6PD, a pivotal enzyme of the PPP, exhibits aberrant overexpression in diverse chemoresistant cancer cells, including ovarian and NSCLC, and confers resistance to oxidative stress and increased viability.^35^, ^36^, ^38^, ^39^, ^40^, ^41^, ^42^ Many previous studies support that an increased level of G6PD could be a hallmark of cisplatin resistance in lung cancer, which is in line with our data (G6PD; logFC: 2.901803, *p.value*: 1.82E‐14).^35^, ^41^, ^42^, ^43^, ^44^ Pyruvate dehydrogenase kinase 1 (PDK1) is an enzyme that inhibits the entry of pyruvate into the TCA cycle, thereby promoting glycolysis and epithelial‐mesenchymal transition (EMT).^45^, ^46^ Previous studies have shown that both G6PD and PDK1 are associated with cisplatin resistance in lung cancer and that their inhibition or downregulation can sensitize cancer cells to cisplatin treatment.^37^, ^46^, ^47^, ^48^ Our findings are consistent with these reports and suggest that G6PD and PDK1 (logFC: 2.114258, *p.value*: 1.19E‐06) are potential targets for overcoming cisplatin resistance in NSCLC.

Our results also showed an increased expression of *NTRK1* in cisplatin‐resistant NSCLC cells compared to sensitive cells (logFC: 2.00152, *p.value*: 1.15E‐09). Neurotrophic tropomyosin receptor kinase 1 (*NTRK1*) encodes TRKA, a tyrosine kinase receptor that mediates neurotrophin signaling and neural development.^49^, ^50^ *TRKA* is overexpressed in various cancers including pancreas, breast, lung, glioblastoma, lymphoid, oral squamous cell carcinoma, and adenoid cystic carcinoma and confers drug resistance and poor prognosis.^51^, ^52^ The neurotrophin signaling pathway is complex and can affect chemoresistance in different ways.^51^, ^52^, ^53^ Moreover, NTRK1 gene fusions are oncogenic and prevalent in some tumors, such as NSCLC, melanoma, glioma, and thyroid cancers.^49^, ^54^ The frequency of NTRK1 fusions in NSCLC is about 0.07%–3.3%.^55^ Targeting NTRK1 fusion proteins have been proposed as a promising strategy to overcome NTRK1‐mediated drug resistance in NSCLC.^56^, ^57^, ^58^, ^59^, ^60^ These evidences are in agreement with our findings and suggest NTRK1 as a potential target of cisplatin resistance in NSCLC, although further studies are needed on this gene in lung cancer.

The GO enrichment analysis revealed that the genes *YTHDF2*, *HEY1*, *PDCD10*, *MAGEA1* might be involved in the chemoresistance of NSCLC cells through the Notch signaling pathway (GO:0008593). The Notch signaling pathway regulates various cellular processes, such as proliferation, survival, and EMT, that enhances tumor aggressiveness and invasiveness.^61^, ^62^, ^63^, ^64^, ^65^

In lung cancer, the Notch signaling pathway maintains the properties of cancer stem cells (CSCs) and confers drug resistance.^66^ CSCs are responsible for therapy resistance and tumor recurrence, as they can evade the effects of chemotherapy, radiotherapy, and immunotherapy. They can also modulate the tumor immune microenvironment (TIME) to create an immunosuppressive niche that protects them from immune attack.^67^

Our result also showed a significant upregulation of *YTHDF2* (logFC: 2.022421721, *p.value*: 1.43E‐12) in cisplatin‐resistant NSCLC cells. YTHDF2 is a major m6A reader that recognizes and binds to m6A‐modified RNAs, a type of epigenetic modification that affects RNA stability and translation.^68^

m6A modulators can affect the CSC phenotype and function by altering the expression and activity of key genes and pathways involved in stemness, epithelial‐mesenchymal transition (EMT), DNA damage response (DDR), autophagy, and immune evasion.^69^ YTHDF2 can have different effects on cancer therapy resistance depending on the type and context of the cancer.^68^ For example, in lung cancer, YTHDF2 promotes cancer progression and resistance to Erlotinib therapy through activating the Notch signaling pathway.^70^

In leukemia, YTHDF2 supports the survival and growth of leukemia stem cells (LSCs) by enhancing the translation of genes that are important for stemness and proliferation, such as MYC and BCL2.^71^ In hepatocellular carcinoma (HCC), YTHDF2 has a dual role: it increases the stemness and tumor growth of liver cancer stem cells (LCSCs) by increasing the translation of OCT4, but it also inhibits the proliferation and growth of HCC cells by decreasing the stability of EGFR.^72^

Conversely, the expression of YTHDF2 affects the sensitivity of melanoma cells to immunotherapy by regulating the level of PD‐1, an immune checkpoint protein.^73^ YTHDF2 promotes the degradation of PD‐1 mRNA, leading to lower PD‐1 protein expression on the surface of melanoma cells.^73^ As a result, YTHDF2 knockdown could increase the resistance of melanoma cells to immunotherapy. This is in line with the observation that high expression of PD1 and PD‐L1 is associated with cisplatin resistance in small cell lung cancer (SCLC) cell lines (H69R, H82R), relative to their parental counterparts.^74^ It has been proposed that intracellular PD1/PD‐L1 signaling may be a determinant of poor response to cisplatin treatment, and that blocking this pathway may enhance the chemosensitivity of aggressive SCLC.

Based on our finding that *YTHDF2* is overexpressed in cisplatin‐resistant cells, we suggest that YTHDF2 may confer drug resistance to lung CSCs by increasing the expression of OCT4, which activates the expression of drug efflux transporters and anti‐apoptotic genes.^75^, ^76^, ^77^ However, we also found that the expression of *CD274*, also known as *PD‐L1*, was decreased in resistant cells compared with sensitive cells (logFC: −5.89, *p.value*: 1.78E‐15). This contradicts the previous studies that showed that *PD‐L1* expression is associated with poor response to immune checkpoint inhibitors in NSCLC patients.

Our results are consistent with the study by Tsuchiya et al., who reported that YTHDF1 and YTHDF2 were associated with a better prognosis and an inflamed tumor‐immune microenvironment in NSCLC by regulating the expression of PD‐1 and PD‐L1.^78^ These findings challenge the notion that CD274 is a marker for poor response to cisplatin treatment in lung cancer and suggest that cisplatin‐resistant NSCLC cells may have developed other mechanisms to evade the immune system. Further studies are needed to elucidate the function of YTHDF and CD274 in the tumor microenvironment and the m6A‐mediated control of transcripts and proteins.

The transcription factor HEY1 is recognized as a key player in the EMT‐mediated decline in survival rates among cancer patients, with its expression indicating the activation of the Notch signaling pathway.^79^, ^80^, ^81^, ^82^ It has been found to be linked with cancer stem cells (CSCs) in a variety of cancers, including glioblastoma, breast cancer, and lung cancer.^83^ HEY1 is known to influence the self‐renewal, survival, and resistance of CSCs by suppressing the expression of genes involved in apoptosis, cell cycle arrest, differentiation, and senescence.^82^ For instance, in glioblastoma stem cells (GSCs), HEY1 is upregulated by the STAT3/NF‐κB signaling pathway, which is constitutively activated in these cells. HEY1, in turn, helps to maintain the stemness and self‐renewal of GSCs by repressing the expression of CTBP1 and RBPJ, which are negative regulators of the Notch pathway.^84^ Recent studies have reported high levels of *HEY1* expression in cisplatin‐resistant lung adenocarcinoma tissues and A549/DDP cell lines.^80^

PDK1 appears to act as a dual‐directional effector in initiating the EMT and inducing cisplatin resistance. The positive role of the PDK1/Notch axis in promoting metastasis through EMT activation in Hypopharyngeal squamous cell carcinoma (HSCC) has been recently emphasized.^85^ Therefore, alongside its role in energy metabolism and cell survival, PDK1 could be a key regulator of the Notch1 signaling pathway in NSCLC. However, the protein expression level of PDK1 in two NSCLC cell lines (H358 and H520) remains unobserved and warrants further investigation.^79^ As a result, based on the existing evidence, it seems that PDK1's impact on drug resistance in NSCLC is part of a multifaceted network of factors, requiring in‐depth laboratory analysis for a complete understanding.

In contrast to PDK1, MAGEA1 seems to exert a negative influence on the Notch signaling pathway within cells. MAGEA1 is part of the *MAGEA* gene family, and the exact functional role of MAGEA proteins is yet to be determined. It is reported that MAGE‐A proteins exhibit varying activity depending on their subcellular localization. MAGE‐A1 has been shown to interfere with Notch as a potent transcriptional repressor by inhibiting the intracellular transactivation domain of Notch1. Moreover, forced expression of MAGEA1 is known to increase the drug sensitivity of cisplatin‐resistant ovarian cancer cells due to induced epigenetic changes.^86^, ^87^ Our findings suggest that the downregulated expression of *MAGEA1* (logFC: −3.8555, *p.value*: 8.44E‐16) in cisplatin‐resistant cells could reduce the inhibitory effects of MAGEA1 on Notch1, leading to cell survival and endurance of resistant cells.

PDCD10, a protein that interacts with various molecules, plays a crucial role in regulating numerous biological processes. The role of PDCD10 in cell survival and proliferation has been confirmed in various types of cancer, including NSCLC, bladder cancer, ovarian cancer, cervical cancer, and prostate cancer.^88^ However, it is demonstrated that the expression of *PDCD10* vary based on the specific type of cancer cell and the anti‐cancer drug utilized. For instance, in colon cancer, *PDCD10* is upregulated, protecting cancer cells from cisplatin‐induced apoptosis.^88^ Conversely, in breast cancer, *PDCD10* is downregulated, making cancer cells more resistant to other anti‐cancer drugs, such as doxorubicin, docetaxel, and etoposide.^89^

In addition to its role in cancer, PDCD10 also influences the Notch signaling pathway a critical regulator of cellular destiny, differentiation, and intercellular communication. This regulation may impact the proliferation, migration, and angiogenesis of endothelial cells, potentially leading to conditions like Cerebral cavernous malformation (CCM) when PDCD10 is lost in these cells.^88^

This highlights the complex and significant relationship between PDCD10 and Notch signaling in various diseases. Our research reveals a decrease in the levels of *PDCD10* (logFC: −3.717, *p.value*: 6.65E‐16) in cisplatin‐resistant NSCLC cells compared to those that are sensitive, highlighting the need for additional experimental investigations for a more thorough comprehension.

The findings of this study were derived through a comprehensive examination of high‐throughput studies, utilizing in silico tools to predict the molecular mechanisms underlying cisplatin resistance. The results obtained provide a foundation upon which researchers can formulate hypotheses for future experimental studies in the field of drug resistance and targeted therapy. Nonetheless, this study does present certain limitations that must be acknowledged.

First, our study focused on specific aspects of the cisplatin resistance in NSCLC, and there may be other relevant factors that were not considered in our analysis. Given the multifaceted characteristics of NSCLC, as well as the multifactorial nature of cisplatin resistance in cancer cells, such as the initiation of anti‐apoptotic signals, active drug expulsion from the cell cytoplasm, miRNA‐mediated epigenetic regulation, growth regulatory pathway deregulation leading to growth factor independence, immune system suppression, and low antigen expression that activates T lymphocyte cells (mimicry)—this study has concentrated on several of these elements.^90^ These include the activity of gap junctions, DNA repair mechanisms, and the deregulation of growth regulatory pathways.

Second, our research did not directly investigate the interaction between the NOTCH pathway and cancer stem cells (CSCs), which have been identified as key players in chemo‐ and radiotherapy resistance in NSCLC. Previous studies have shown that CSCs, particularly the side population (SP) cells in the A549 cell line, possess increased proliferation, higher clonogenicity, stronger tumorigenicity, and resistance to chemotherapy.^91^, ^92^ However, our current analysis did not specifically evaluate the expression of CSC‐associated genes or their potential impact on treatment resistance. This represents an area for future research, which could provide a more comprehensive understanding of the complex interplay between the NOTCH pathway, CSCs, and treatment resistance in NSCLC.

Third, our study was purely computational and did not include any in vitro or in vivo experiments to validate the findings. Therefore, the results should be interpreted with caution until validated experimentally.

## 5 CONCLUSION

We have discovered several molecular determinants of cisplatin resistance in NSCLC cell lines, involving genes and pathways related to gap junction communication, DNA damage response, ROS modulation, EMT induction, and stemness maintenance. We have also revealed the potential therapeutic value of targeting *SMUG1*, *NTHL1*, *PARP3*, *NTRK1*, *YTHDF2*, *PDK1*, *CD274*, *HEY1*, *PDCD10* and *MAGEA1* which are differentially expressed in cisplatin‐resistant NSCLC cells.

Our study sheds light on the complex mechanisms underlying cisplatin resistance and offers novel opportunities for improving the treatment outcomes of NSCLC. Nevertheless, further investigations are required to confirm the clinical utility and biological importance of these genes and pathways in NSCLC patients. Elucidating the precise role of candidate DEGs and pathways associated with drug resistance in the cancer cell metabolism and survival could facilitate the clinical management and pave the way for the development of personalized medicine.

## AUTHOR CONTRIBUTIONS

**Somayeh Hashemi Sheikhshabani:** Conceptualization (equal); funding acquisition (equal); investigation (equal); methodology (equal); project administration (equal); writing – original draft (equal). **Paratoo Modarres:** Software (equal); validation (equal); writing – original draft (equal). **Soudeh Ghafouri‐Fard:** Writing – original draft (equal); writing – review and editing (equal). **Zeinab Amini‐Farsani:** Investigation (equal); methodology (equal); writing – original draft (lead). **Lavin Khodaee:** Data curation (equal); formal analysis (equal). **Nasibeh Shaygan:** Data curation (equal); formal analysis (equal). **Zahra Amini‐Farsani:** Software (equal). **Mir Davood Omrani:** Methodology (equal); project administration (equal).

## CONFLICT OF INTEREST STATEMENT

The authors declare no conflicts of interest.

## ETHICS STATEMENT

Not applicable.

## Supporting information


**Table S1.** Gene ontology terms in biological process group for DEGs related to NSCLC cisplatin‐resistance provided by Enrichr based on *p*‐value ranking for each category.

**Table S2.** Gene ontology terms in cellular component group for DEGs related to NSCLC cisplatin‐resistance provided by Enrichr based on *p*‐value ranking for each category.

**Table S3.** Gene ontology terms in molecular function group for DEGs related to NSCLC cisplatin‐resistance provided by Enrichr based on *p*‐value ranking for each category.

## Data Availability

The sequencing data used in this work are available in the Gene Expression Omnibus (GEO) accession numbers GSE108214, GSE84146, and GSE21656.
