# Supplementary material for: Meta‐analysis of microarray data to determine gene indicators involved in cisplatin resistance in non‐small cell lung cancer
Source: Cancer Rep (Hoboken). 2024 Feb 13;7(2):e1970. doi: 10.1002/cnr2.1970 (PMC10864718; doi:10.1002/cnr2.1970)
Supplement: Supplementary file 1 — Table S1. Gene ontology terms in biological process group for DEGs related to NSCLC cisplatin‐resistance provided by Enrichr based on p‐value ranking for each category. [file CNR2-7-e1970-s001.docx]

| Term | P-value | Genes |
| --- | --- | --- |
| regulation of primary metabolic process (GO:0080090) | 4.31E-04 | IDI1;PSMD12;PSMC3;NFYB;SCD;PMVK;ODC1;CERS2 |
| regulation of Notch signaling pathway (GO:0008593) | 9.78E-04 | YTHDF2;HEY1;PDCD10;MAGEA1;TSPAN5;SYNJ2BP |
| cellular macromolecule biosynthetic process (GO:0034645) | 0.001379363 | RPS15;MRPL42;KIN;TNIP1;HUS1;CREBL2;NAP1L1;RPL15;RPS11;RPS4Y1;LARGE1;POLR2I |
| regulation of p38MAPK cascade (GO:1900744) | 0.001594784 | DUSP10;GADD45A;ZC3H12A;MINK1 |
| phospholipid biosynthetic process (GO:0008654) | 0.001594784 | DPAGT1;IDI1;AGK;CPNE3 |
| isopentenyl diphosphate biosynthetic process (GO:0009240) | 0.00181975 | IDI1;PMVK |
| cotranslational protein targeting to membrane (GO:0006613) | 0.00186199 | RPS15;SEC61A1;RPL15;RPS11;RPS4Y1;SIL1 |
| glycolipid biosynthetic process (GO:0009247) | 0.002137077 | PGAP4;PIGM;LARGE1;PIGH |
| regulation of protein localization to cilium (GO:1903564) | 0.002704962 | CCDC88A;CCDC66 |
| cellular response to glucose starvation (GO:0042149) | 0.0027948 | SESN3;ZC3H12A;SIK1;ATG14 |
| negative regulation of response to wounding (GO:1903035) | 0.002817639 | CLDN3;CRK;CERS2 |
| positive regulation of mRNA catabolic process (GO:0061014) | 0.003041637 | NANOS1;YTHDF2;ZC3H12A;TNRC6B |
| negative regulation of epithelial cell proliferation (GO:0050680) | 0.003082728 | TGFBR3;DUSP10;NUPR1;NR2F2;SYNJ2BP |
| purine-containing compound metabolic process (GO:0072521) | 0.003752768 | MACROD1;FHIT |
| cellular response to laminar fluid shear stress (GO:0071499) | 0.003752768 | MAPK7;MAP2K5 |
| regulation of histamine secretion by mast cell (GO:1903593) | 0.003752768 | SNX4;VAMP7 |
| regulation of protein deubiquitination (GO:0090085) | 0.003752768 | TNIP1;ZC3H12A |
| heart trabecula formation (GO:0060347) | 0.003752768 | TGFBR3;HEY1 |
| positive regulation of protein deubiquitination (GO:1903003) | 0.003752768 | TNIP1;ZC3H12A |
| positive regulation of p38MAPK cascade (GO:1900745) | 0.004160088 | GADD45A;MINK1;ZC3H12A |
| regulation of protein metabolic process (GO:0051246) | 0.004177727 | MAPK7;ODC1;SPOPL;MAP2K5 |
| depyrimidination (GO:0045008) | 0.004958578 | SMUG1;NTHL1 |
| DNA modification (GO:0006304) | 0.005237442 | SMUG1;PARP3;NTHL1 |
| signal transduction in response to DNA damage (GO:0042770) | 0.005570357 | GADD45A;TIPRL;HUS1;INTS7 |
| regulation of CD8-positive, alpha-beta T cell activation (GO:2001185) | 0.006317899 | CD274;IRF1 |
| glycerolipid biosynthetic process (GO:0045017) | 0.006317899 | AGK;CPNE3 |
| positive regulation of mast cell degranulation (GO:0043306) | 0.006317899 | SNX4;VAMP7 |
| carbohydrate derivative biosynthetic process (GO:1901137) | 0.006467788 | G6PD;TNIP1;LARGE1 |
| GPI anchor metabolic process (GO:0006505) | 0.006467788 | PGAP4;PIGM;PIGH |
| positive regulation of proteasomal protein catabolic process (GO:1901800) | 0.006576702 | ZER1;PSMC3;RNF19B;NUPR1;HSPBP1 |
| ncRNA processing (GO:0034470) | 0.006658571 | RPS15;POP5;SMAD1;IMP3;DIS3;PES1;INTS7;RPL15 |
| Rap protein signal transduction (GO:0032486) | 0.007826329 | RAP2B;PLK2 |
| regulation of cell-cell adhesion involved in gastrulation (GO:0070587) | 0.007826329 | MAPK7;MAP2K5 |
| response to laminar fluid shear stress (GO:0034616) | 0.007826329 | MAPK7;MAP2K5 |
| negative regulation of heterotypic cell-cell adhesion (GO:0034115) | 0.007826329 | MAPK7;MAP2K5 |
| establishment of spindle orientation (GO:0051294) | 0.007856264 | MOS;NDC80;CDK5RAP2 |
| negative regulation of Notch signaling pathway (GO:0045746) | 0.007856264 | YTHDF2;HEY1;MAGEA1 |
| SRP-dependent cotranslational protein targeting to membrane (GO:0006614) | 0.007942917 | RPS15;SEC61A1;RPL15;RPS11;RPS4Y1 |
| regulation of lipid metabolic process (GO:0019216) | 0.008694923 | IDI1;NFYB;SCD;PMVK;CERS2 |
| DNA damage checkpoint signaling (GO:0000077) | 0.009407238 | TIPRL;HUS1;INTS7 |
| GPI anchor biosynthetic process (GO:0006506) | 0.009407238 | PGAP4;PIGM;PIGH |
| negative regulation of response to cytokine stimulus (GO:0060761) | 0.009479558 | MAPK7;MAP2K5 |
| rRNA processing (GO:0006364) | 0.009974903 | RPS15;POP5;IMP3;DIS3;PES1;SRFBP1;RPL15 |
| actin cytoskeleton reorganization (GO:0031532) | 0.0109069 | SIPA1L1;CLDN3;MINK1;MYH9 |
| autophagosome maturation (GO:0097352) | 0.011124353 | MAP1LC3A;VAMP7;ATG14 |
| secondary alcohol biosynthetic process (GO:1902653) | 0.011124353 | IDI1;G6PD;PMVK |
| negative regulation of blood vessel endothelial cell migration (GO:0043537) | 0.011124353 | PDCD10;GADD45A;MAP2K5 |
| cellular response to fluid shear stress (GO:0071498) | 0.011273366 | MAPK7;MAP2K5 |
| intrinsic apoptotic signaling pathway in response to oxidative stress (GO:0008631) | 0.011273366 | PDCD10;PDK1 |
| negative regulation by host of viral process (GO:0044793) | 0.011273366 | PSMC3;ZC3H12A |
| regulation of cilium assembly (GO:1902017) | 0.011511678 | IFT20;CCDC88A;TBC1D2B;ARHGAP35 |
| positive regulation of stress-activated MAPK cascade (GO:0032874) | 0.011712898 | PDCD10;GADD45A;ZC3H12A;MINK1;TRAF5 |
| cholesterol biosynthetic process (GO:0006695) | 0.012046166 | IDI1;G6PD;PMVK |
| negative regulation of epithelial cell migration (GO:0010633) | 0.012046166 | DUSP10;NR2F2;SYNJ2BP |
| positive regulation of interferon-beta production (GO:0032728) | 0.013010575 | OAS1;IRF1;DHX58 |
| purine-containing compound salvage (GO:0043101) | 0.013203618 | GMPR;AMPD2 |
| pyrimidine deoxyribonucleotide catabolic process (GO:0009223) | 0.013203618 | SMUG1;NTHL1 |
| regulation of chemokine (C-X-C motif) ligand 2 production (GO:2000341) | 0.013203618 | OAS1;MAP2K5 |
| regulation of mitotic cell cycle spindle assembly checkpoint (GO:0090266) | 0.013203618 | NDC80;CDK5RAP2 |
| kinetochore organization (GO:0051383) | 0.013203618 | CENPT;NDC80 |
| negative regulation of stem cell differentiation (GO:2000737) | 0.013203618 | YTHDF2;TCF15 |
| protein targeting to ER (GO:0045047) | 0.013723021 | RPS15;SEC61A1;RPL15;RPS11;RPS4Y1 |
| negative regulation of endothelial cell proliferation (GO:0001937) | 0.014017854 | PDCD10;NR2F2;SYNJ2BP |
| plasma membrane bounded cell projection assembly (GO:0120031) | 0.01480976 | IFT20;CCDC88A;TTC8;RAP2B;RP2;MINK1;SEPTIN6;CCDC66;RHOD |
| regulation of JNK cascade (GO:0046328) | 0.014810086 | TGFBR3;DUSP10;GADD45A;MINK1;TRAF5 |
| sterol biosynthetic process (GO:0016126) | 0.015068236 | IDI1;G6PD;PMVK |
| negative regulation of endothelial cell migration (GO:0010596) | 0.015068236 | GADD45A;NR2F2;SYNJ2BP |
| negative regulation of innate immune response (GO:0045824) | 0.015068236 | YTHDF2;OAS1;DHX58 |
| microvillus organization (GO:0032528) | 0.015266268 | RAP2B;MINK1 |
| negative regulation of cell migration involved in sprouting angiogenesis (GO:0090051) | 0.015266268 | PDCD10;MAP2K5 |
| DNA integrity checkpoint signaling (GO:0031570) | 0.01616192 | TIPRL;HUS1;INTS7 |
| regulation of hematopoietic stem cell differentiation (GO:1902036) | 0.01635067 | PSMD12;YTHDF2;TCF15;PSMC3 |
| regulation of nuclear-transcribed mRNA catabolic process, deadenylation-dependent decay (GO:1900151) | 0.017457353 | NANOS1;TNRC6B |
| microvillus assembly (GO:0030033) | 0.017457353 | RAP2B;MINK1 |
| negative regulation of cellular component movement (GO:0051271) | 0.017457353 | TGFBR3;CRK |
| positive regulation of nuclear-transcribed mRNA catabolic process, deadenylation-dependent decay (GO:1900153) | 0.017457353 | NANOS1;TNRC6B |
| regulation of actin filament-based process (GO:0032970) | 0.017934507 | CCDC88A;RHOD;CRK;ARHGAP35 |
| response to growth factor (GO:0070848) | 0.018479823 | MAPK7;CPNE3;MAP2K5 |
| epiboly involved in wound healing (GO:0090505) | 0.019772993 | PDCD10;ARHGAP35 |
| sympathetic nervous system development (GO:0048485) | 0.019772993 | NTRK1;FZD3 |
| N-acetylglucosamine metabolic process (GO:0006044) | 0.019772993 | GNPDA2;LARGE1 |
| negative regulation of type I interferon-mediated signaling pathway (GO:0060339) | 0.019772993 | YTHDF2;OAS1 |
| negative regulation of cell motility (GO:2000146) | 0.020413869 | CLDN3;DUSP10;RAP2B;PIP5KL1;CRK |
| positive regulation of protein modification by small protein conjugation or removal (GO:1903322) | 0.021380423 | TNIP1;ZC3H12A;UBE3A;HSPBP1 |
| copper ion homeostasis (GO:0055070) | 0.022209392 | ANKRD9;CUTC |
| establishment of protein localization to endoplasmic reticulum (GO:0072599) | 0.022209392 | RYR2;SEC61A1 |
| snRNA metabolic process (GO:0016073) | 0.022209392 | INTS7;RBM7 |
| negative regulation of chemokine production (GO:0032682) | 0.022209392 | OAS1;MAP2K5 |
| preassembly of GPI anchor in ER membrane (GO:0016254) | 0.022209392 | PIGM;PIGH |
| mRNA processing (GO:0006397) | 0.022993389 | ERN1;ISY1;HNRNPM;KIN;DDX39A;PHF5A;POLR2I;PRPF8;CTNNBL1 |
| negative regulation of response to external stimulus (GO:0032102) | 0.02329517 | CLDN3;MAPK7;DHX58;CRK;CERS2 |
| cellular response to radiation (GO:0071478) | 0.023640327 | GADD45A;HUS1;INTS7 |
| glycerophospholipid metabolic process (GO:0006650) | 0.024213204 | AGK;CPNE3;PIGM;PIGH |
| rRNA metabolic process (GO:0016072) | 0.024375841 | RPS15;POP5;IMP3;DIS3;PES1;RPL15 |
| peptide biosynthetic process (GO:0043043) | 0.024375841 | RPS15;MRPL42;TNIP1;RPL15;RPS11;RPS4Y1 |
| protein O-linked mannosylation (GO:0035269) | 0.02476283 | SDF2L1;LARGE1 |
| ventricular cardiac muscle cell action potential (GO:0086005) | 0.02476283 | KCNH2;RYR2 |
| posttranscriptional regulation of gene expression (GO:0010608) | 0.025039947 | NANOS1;ZC3H7B;ATG14 |
| negative regulation of protein modification process (GO:0031400) | 0.025205479 | G6PD;PIP5KL1;ZC3H12A;ATG14 |
| cellular protein modification process (GO:0006464) | 0.025340258 | NTRK1;PARP3;PSMD12;YES1;EPAS1;PLK2;MINK1;ANKRD9;ERN1;DUSP10;PSMC3;BTBD1;STK17A;QPCT;B3GNT3;MACROD1;SIK1;SGK3;LARGE1;PDK1;CDK16;PIGH |
| gene expression (GO:0010467) | 0.025484979 | RPS15;MRPL42;DDX39A;TNIP1;TSPAN5;CREBL2;RPL15;RPS11;RPS4Y1;POLR2I |
| regulation of mRNA catabolic process (GO:0061013) | 0.026427237 | PSMD12;YTHDF2;PSMC3;DIS3;ZC3H12A |
| regulation of centriole replication (GO:0046599) | 0.027429668 | PLK2;CDK5RAP2 |
| establishment or maintenance of apical/basal cell polarity (GO:0035088) | 0.027429668 | DLG2;SYNJ2BP |
| heart trabecula morphogenesis (GO:0061384) | 0.027429668 | TGFBR3;HEY1 |
| positive regulation of cilium assembly (GO:0045724) | 0.027429668 | CCDC88A;ARHGAP35 |
| positive regulation of apoptotic process (GO:0043065) | 0.027621277 | TIAM2;GADD45A;STK17A;PIP5KL1;ZC3H12A;SIK1;NUPR1;CTSD;CTNNBL1 |
| cellular response to ionizing radiation (GO:0071479) | 0.027970356 | GADD45A;HUS1;INTS7 |
| non-canonical Wnt signaling pathway (GO:0035567) | 0.028089268 | PSMD12;FZD3;DAAM1;PSMC3;TNRC6B |
| translation (GO:0006412) | 0.028438104 | RPS15;MRPL42;TNIP1;RPL15;RPS11;RPS4Y1;MRPL57 |
| Wnt signaling pathway, planar cell polarity pathway (GO:0060071) | 0.029417518 | PSMD12;FZD3;DAAM1;PSMC3 |
| regulation of interferon-beta production (GO:0032648) | 0.029500976 | OAS1;IRF1;DHX58 |
| positive regulation of reactive oxygen species metabolic process (GO:2000379) | 0.029500976 | GADD45A;ZC3H12A;RAB27A |
| base-excision repair, AP site formation (GO:0006285) | 0.030206344 | SMUG1;NTHL1 |
| cardiac epithelial to mesenchymal transition (GO:0060317) | 0.030206344 | TGFBR3;HEY1 |
| cellular response to interferon-beta (GO:0035458) | 0.030206344 | OAS1;IRF1 |
| nucleobase-containing compound biosynthetic process (GO:0034654) | 0.030206344 | GMPR;AMPD2 |
| positive regulation of hormone secretion (GO:0046887) | 0.030206344 | SNX4;VAMP7 |
| positive regulation of nitrogen compound metabolic process (GO:0051173) | 0.030206344 | MAPK7;MAP2K5 |
| negative regulation of phosphorylation (GO:0042326) | 0.030531705 | PIP5KL1;ZC3H12A;NUPR1;ATG14 |
| negative regulation of ERK1 and ERK2 cascade (GO:0070373) | 0.031075054 | DUSP10;TNIP1;SYNJ2BP |
| regulation of transcription from RNA polymerase II promoter in response to stress (GO:0043618) | 0.031670513 | PSMD12;MAPK7;PSMC3;EPAS1 |
| regulation of axonogenesis (GO:0050770) | 0.032692445 | TIAM2;SIPA1L1;ARHGAP35 |
| regulation of cellular amine metabolic process (GO:0033238) | 0.032692445 | PSMD12;PSMC3;ODC1 |
| mitotic metaphase plate congression (GO:0007080) | 0.032692445 | KIF18A;CHMP7;NDC80 |
| regulation of establishment of planar polarity (GO:0090175) | 0.032833999 | PSMD12;FZD3;DAAM1;PSMC3 |
| calcium-mediated signaling using intracellular calcium source (GO:0035584) | 0.033089372 | RYR2;ADGRL1 |
| cyclic purine nucleotide metabolic process (GO:0052652) | 0.033089372 | AMPD2;ADCY1 |
| regulation of transcription by RNA polymerase III (GO:0006359) | 0.033089372 | ZNF143;PRDX5 |
| positive regulation of inflammatory response (GO:0050729) | 0.034022217 | SNX4;VAMP7;TNIP1;NUPR1 |
| positive regulation of transcription, DNA-templated (GO:0045893) | 0.035568939 | KCNH2;ZNF143;SMAD1;TCF15;ONECUT2;EPAS1;NFYB;ZBTB48;CXXC1;PBX2;CREBL2;NR2F2;MED12;PHF5A;MAPK7;HEY1;PSMC3;TNIP1;IRF1;ZC3H12A;HOXB3;FIZ1;MAP2K5;CDK5RAP2 |
| mRNA splicing, via spliceosome (GO:0000398) | 0.035644658 | ISY1;HNRNPM;DDX39A;PHF5A;POLR2I;PRPF8;SNUPN;CTNNBL1 |
| cardiac muscle tissue development (GO:0048738) | 0.036056482 | SMAD1;HEY1;SIK1 |
| heart morphogenesis (GO:0003007) | 0.036056482 | TGFBR3;RYR2;HEY1 |
| IRE1-mediated unfolded protein response (GO:0036498) | 0.036056482 | ERN1;KLHDC3;CXXC1 |
| protein mannosylation (GO:0035268) | 0.036075338 | SDF2L1;LARGE1 |
| regulation of execution phase of apoptosis (GO:1900117) | 0.036075338 | FZD3;ZC3H12A |
| regulation of response to cytokine stimulus (GO:0060759) | 0.036075338 | MAPK7;MAP2K5 |
| negative regulation of biomineral tissue development (GO:0070168) | 0.036075338 | ECM1;HEY1 |
| positive regulation of neural precursor cell proliferation (GO:2000179) | 0.036075338 | FZD3;NAP1L1 |
| regulation of actin cytoskeleton organization (GO:0032956) | 0.036473012 | CCDC88A;RHOD;CRK;ARHGAP35 |
| regulation of stem cell differentiation (GO:2000736) | 0.036473012 | PSMD12;YTHDF2;TCF15;PSMC3 |
| regulation of cellular amino acid metabolic process (GO:0006521) | 0.037802734 | PSMD12;PSMC3;ODC1 |
| mitotic cell cycle checkpoint signaling (GO:0007093) | 0.039160905 | PLK2;ZFYVE19 |
| negative regulation of extrinsic apoptotic signaling pathway in absence of ligand (GO:2001240) | 0.039160905 | MAPK7;MAP2K5 |
| negative regulation of signal transduction in absence of ligand (GO:1901099) | 0.039160905 | MAPK7;MAP2K5 |
| positive regulation of transcription initiation from RNA polymerase II promoter (GO:0060261) | 0.039160905 | MED12;PSMC3 |
| telomere maintenance (GO:0000723) | 0.041422568 | PARP3;ZBTB48;HUS1 |
| mRNA metabolic process (GO:0016071) | 0.041672781 | ERN1;KIN;YTHDF2;PRPF8 |
| establishment of mitotic spindle orientation (GO:0000132) | 0.042342803 | NDC80;CDK5RAP2 |
| wound healing, spreading of cells (GO:0044319) | 0.042342803 | PDCD10;ARHGAP35 |
| potassium ion homeostasis (GO:0055075) | 0.042342803 | KCNH2;SLC12A7 |
| protein alpha-1,2-demannosylation (GO:0036508) | 0.042342803 | EDEM3;DERL2 |
| regulation of reactive oxygen species metabolic process (GO:2000377) | 0.043295639 | GADD45A;STK17A;ZC3H12A |
| negative regulation of DNA recombination (GO:0045910) | 0.043295639 | PARP3;FBH1;POLR2I |
| cAMP-mediated signaling (GO:0019933) | 0.045617835 | MAPK7;ADCY1 |
| cardiac conduction system development (GO:0003161) | 0.045617835 | SMAD1;HEY1 |
| regulation of heterotypic cell-cell adhesion (GO:0034114) | 0.045617835 | MAPK7;MAP2K5 |
| negative regulation of cellular carbohydrate metabolic process (GO:0010677) | 0.045617835 | SIK1;NUPR1 |
| antigen processing and presentation of peptide antigen via MHC class II (GO:0002495) | 0.048731876 | KIF18A;SEC23A;MARCHF8;CTSD |
| purine-containing compound biosynthetic process (GO:0072522) | 0.048982874 | GMPR;AMPD2 |
| regulation of biomineral tissue development (GO:0070167) | 0.048982874 | ECM1;HEY1 |
| regulation of regulatory T cell differentiation (GO:0045589) | 0.048982874 | DUSP10;IRF1 |
| lamellipodium assembly (GO:0030032) | 0.048982874 | CCDC88A;RHOD |
| TOR signaling (GO:0031929) | 0.048982874 | CCDC88A;TIPRL |
| ribosome biogenesis (GO:0042254) | 0.04921344 | RPS15;POP5;IMP3;DIS3;PES1;RPL15 |
| phosphorylation (GO:0016310) | 0.049710138 | ERN1;NTRK1;AGK;STK17A;PLK2;MINK1;SIK1;SGK3;PDK1;CDK16 |
