# Supplementary material for: Meta‐analysis of microarray data to determine gene indicators involved in cisplatin resistance in non‐small cell lung cancer
Source: Cancer Rep (Hoboken). 2024 Feb 13;7(2):e1970. doi: 10.1002/cnr2.1970 (PMC10864718; doi:10.1002/cnr2.1970)
Supplement: Supplementary file 2 — Table S2. Gene ontology terms in cellular component group for DEGs related to NSCLC cisplatin‐resistance provided by Enrichr based on p‐value ranking for each category. [file CNR2-7-e1970-s002.docx]

| Term | P-value | Genes |
| --- | --- | --- |
| P-body (GO:0000932) | 8.06E-04 | YTHDF2;BTBD1;PSMC3;ZC3H12A;SYNE1;TNRC6B |
| endoplasmic reticulum membrane (GO:0005789) | 0.004499699 | DPAGT1;RYR2;SEC23A;SDF2L1;DERL2;MOGS;ATG14;COPZ1;PLD2;CTAGE1;ERN1;SEC61A1;VAMP7;RDH11;SCD;ZC3H12A;PIGM;PIGH;CERS2 |
| endosome membrane (GO:0010008) | 0.005401643 | NTRK1;SNX4;CD274;RAP2B;VAMP7;GGA3;RAB14;MARCHF8;CTSD;RHOD;ATP6V0A1 |
| recycling endosome membrane (GO:0055038) | 0.008190925 | CD274;RAP2B;GGA3;RAB14 |
| post-mRNA release spliceosomal complex (GO:0071014) | 0.009479558 | ISY1;HNRNPM |
| early endosome membrane (GO:0031901) | 0.010787355 | CD274;SNX4;GGA3;RAB14;MARCHF8 |
| U2-type catalytic step 1 spliceosome (GO:0071006) | 0.011273366 | ISY1;PRPF8 |
| microtubule (GO:0005874) | 0.012919914 | KIF18A;TUBB2B;TUBA3D;TUBAL3;CCDC66;TUBB4A;CDK5RAP2 |
| microtubule cytoskeleton (GO:0015630) | 0.016320509 | TUBB2B;TUBA3D;KIF18A;TUBAL3;CDC42EP2;SEPTIN6;CCDC66;TUBB4A;CDK5RAP2;CDK16 |
| cytosolic small ribosomal subunit (GO:0022627) | 0.017299072 | RPS15;RPS4Y1;RPS11 |
| small ribosomal subunit (GO:0015935) | 0.023640327 | RPS15;RPS4Y1;RPS11 |
| intrinsic component of endoplasmic reticulum membrane (GO:0031227) | 0.025620359 | DPAGT1;ERN1;SEC61A1;SCD;DERL2 |
| postsynaptic density membrane (GO:0098839) | 0.030206344 | DLG2;SYNJ2BP |
| nucleus (GO:0005634) | 0.030353927 | ISY1;ONECUT2;FBH1;ARID4A;SYNE1;RPS15;MED12;DUSP10;HEY1;ZC3H12A;MAGEA1;MACROD1;IDH3B;CPNE3;RBM7;CTNNBL1;TGIF2;TCF15;IMP3;HUS1;THAP2;KIN;TUBB2B;DDX39A;OAS1;IRF1;HOXB3;MYH9;SPOPL;SRFBP1;SIK1;FIZ1;NUPR1;CRK;ZNF230;ANKRD16;CXXC1;ZNF22;CREBL2;RPS4Y1;PRPF8;FHIT;PRDX5;MAPK7;TCEAL4;NTHL1;HLTF;RPL15;POLR2I;ZNF222;SMAD1;YTHDF2;GADD45A;NFYB;PLK2;NCAPH2;PBX2;NAP1L1;NR2F2;TUBB4A;NDC80;HNRNPM;KIF18A;CDAN1;PHF5A;PSMC3;ZNF71;GNPDA2;STK17A;PKIA;ANP32E;INTS7;SERGEF;CUTC;CDK16 |
| postsynaptic specialization membrane (GO:0099634) | 0.033089372 | DLG2;SYNJ2BP |
| intracellular membrane-bounded organelle (GO:0043231) | 0.033520978 | DPAGT1;ISY1;ONECUT2;FBH1;ARID4A;SYNE1;RPS15;MED12;MAP1LC3A;DUSP10;HEY1;ZC3H12A;MAGEA1;MACROD1;IDH3B;CPNE3;RBM7;CTNNBL1;G6PD;TGIF2;TCF15;IMP3;HUS1;RHOD;THAP2;KIN;TUBB2B;DDX39A;VAMP7;OAS1;IRF1;HOXB3;MYH9;SPOPL;SRFBP1;SIK1;SGK3;FIZ1;NUPR1;CRK;ZNF230;ANKRD16;CXXC1;ZNF22;CREBL2;RPS4Y1;PRPF8;FHIT;PRDX5;MAPK7;TCEAL4;NTHL1;HLTF;RPL15;POLR2I;ATP6V0A1;ZNF222;SMAD1;YTHDF2;GADD45A;AGK;NFYB;PLK2;NCAPH2;PBX2;NAP1L1;NR2F2;TUBB4A;NDC80;IFT20;HNRNPM;KIF18A;CDAN1;PHF5A;PSMC3;ZNF71;GNPDA2;RAB14;STK17A;PKIA;ANP32E;INTS7;SERGEF;CUTC;CDK16 |
| cytoskeleton (GO:0005856) | 0.037642295 | CD274;ONECUT2;SEPTIN6;DSTN;RHOD;SYN1;TUBB2B;KIF18A;SIPA1L1;ZC3H12A;CDC42EP2;MYH9;CDK5RAP2;CDK16 |
| cytoplasmic side of plasma membrane (GO:0009898) | 0.039591511 | G6PD;TRAF5;MYH9 |
| integral component of endoplasmic reticulum membrane (GO:0030176) | 0.046034294 | DPAGT1;ERN1;SEC61A1;SCD;DERL2 |
| late endosome (GO:0005770) | 0.04622044 | NTRK1;VAMP7;RAB14;MARCHF8;RAB27A;CHMP7 |
