# Supplementary material for: Meta‐analysis of microarray data to determine gene indicators involved in cisplatin resistance in non‐small cell lung cancer
Source: Cancer Rep (Hoboken). 2024 Feb 13;7(2):e1970. doi: 10.1002/cnr2.1970 (PMC10864718; doi:10.1002/cnr2.1970)
Supplement: Supplementary file 3 — Table S3. Gene ontology terms in molecular function group for DEGs related to NSCLC cisplatin‐resistance provided by Enrichr based on p‐value ranking for each category. [file CNR2-7-e1970-s003.docx]

| Term | P-value | Genes |
| --- | --- | --- |
| RNA binding (GO:0003723) | 2.15E-04 | ISY1;POP5;RPS4Y1;PRPF8;SYNE1;RPS15;MRPL42;ZC3H7B;PES1;DHX58;ZC3H12A;HLTF;CPNE3;RPL15;RPS11;ZC3H13;RBM7;NANOS1;DDX59;YTHDF2;TYW5;IMP3;ELP1;NAP1L1;SSRP1;SNUPN;HNRNPM;KIN;DDX39A;PHF5A;OAS1;CCDC9;MYH9;RBM42;SRFBP1;TNRC6B |
| purine ribonucleoside triphosphate binding (GO:0035639) | 2.20E-04 | TUBAL3;MINK1;RAB27A;SEPTIN6;RHOD;TUBB4A;SYN1;ERN1;TUBB2B;TUBA3D;RAP2B;OAS1;RAB14;STK17A;PMVK;MYH9;SIK1 |
| GTP binding (GO:0005525) | 0.001223437 | TUBB2B;TUBA3D;RAP2B;TUBAL3;RAB14;RAB27A;SEPTIN6;RHOD;TUBB4A |
| hydrolase activity, hydrolyzing N-glycosyl compounds (GO:0016799) | 0.001247912 | SMUG1;NTHL1;MACROD1 |
| guanyl ribonucleotide binding (GO:0032561) | 0.002853614 | TUBB2B;TUBA3D;RAP2B;TUBAL3;RAB14;RAB27A;SEPTIN6;RHOD;TUBB4A |
| intramolecular oxidoreductase activity, interconverting aldoses and ketoses (GO:0016861) | 0.004958578 | GNPDA2;MRI1 |
| DNA N-glycosylase activity (GO:0019104) | 0.004958578 | SMUG1;NTHL1 |
| pre-mRNA intronic binding (GO:0097157) | 0.004958578 | RBM7;PRPF8 |
| endonuclease activity (GO:0004519) | 0.005196841 | ERN1;DIS3;NTHL1;ZC3H12A |
| mitogen-activated protein kinase binding (GO:0051019) | 0.007141969 | DUSP10;MAPK7;TNIP1 |
| adenyl nucleotide binding (GO:0030554) | 0.009479558 | SIL1;HSPBP1 |
| adenyl-nucleotide exchange factor activity (GO:0000774) | 0.009479558 | SIL1;HSPBP1 |
| protein kinase binding (GO:0019901) | 0.0103442 | RYR2;SMAD1;RHOD;SYN1;CCDC88A;DUSP10;MAPK7;TNIP1;PDCD10;PKIA;SIK1;SLC12A7;CRK;CDK5RAP2 |
| intramolecular oxidoreductase activity, transposing C=C bonds (GO:0016863) | 0.013203618 | IDI1;ECH1 |
| aldehyde dehydrogenase (NAD+) activity (GO:0004029) | 0.013203618 | ALDH1L2;ADH5 |
| protein kinase A catalytic subunit binding (GO:0034236) | 0.013203618 | RYR2;PKIA |
| myosin V binding (GO:0031489) | 0.017457353 | RAB14;RAB39B |
| protein serine/threonine kinase activity (GO:0004674) | 0.020714103 | MOS;ERN1;MAPK7;STK17A;PLK2;MINK1;CPNE3;SIK1;SGK3;CDK16 |
| ribosome binding (GO:0043022) | 0.027970356 | SEC61A1;ZC3H12A;GTPBP6 |
| ATP binding (GO:0005524) | 0.038300295 | ERN1;OAS1;STK17A;MINK1;PMVK;MYH9;SIK1;SYN1 |
| GTPase regulator activity (GO:0030695) | 0.041994651 | CCDC88A;SEC23A;SIPA1L1;RP2;CDC42EP2;TBC1D2B;ARHGAP35 |
| GTPase activator activity (GO:0005096) | 0.04264305 | CCDC88A;SEC23A;SIPA1L1;DIS3;RP2;CDC42EP2;TBC1D2B;SERGEF;ARHGAP35 |
| transition metal ion binding (GO:0046914) | 0.04365708 | SEC23A;PHF5A;TYW5;SCD;QPCT;DHX58;ZNF22;LARGE1;ADH5;CUTC;THAP11 |
| epidermal growth factor receptor binding (GO:0005154) | 0.048982874 | SNX4;CCDC88A |
| nuclease activity (GO:0004518) | 0.049164048 | DIS3;NTHL1;ZC3H12A |
